# Autophagy and polyglutamine diseases

**DOI:** 10.1016/j.pneurobio.2011.08.013

**Published:** 2012-05

**Authors:** Maria Jimenez-Sanchez, Frances Thomson, Eszter Zavodszky, David C. Rubinsztein

**Affiliations:** Department of Medical Genetics, University of Cambridge, Cambridge Institute for Medical Research, Addenbrooke's Hospital, Hills Road, Cambridge CB2 0XY, UK

**Keywords:** HD, Huntington's disease, SCA, spinocerebellar ataxia, DRPLA, Denatorubral-pallidoluysian atrophy, SBMA, spinal and bulbar muscular atropy, Htt, Huntingtin, UPS, ubiquitin–proteasome system, HDL-2, Huntington's disease-like 2, IBs, inclusion bodies, RNAi, RNA interference, Atg, autophagy-related genes, ER, endoplasmic reticulum, PI3K, phosphatidylinositol 3-kinase, JNK1, c-Jun N-terminal protein kinase 1, PE, phosphatidylethanolamine, SNAREs, soluble N-ethylmaleimide-sensitive factor attachment protein receptors, mTOR, mammalian target of rapamycin, PI-3-P, phosphatidylinositol-3-phosphate, ROS, reactive oxygen species, IP_3_, inositol-1,4,5-triphosphate, IP_3_R, IP_3_ receptors, cAMP, cyclic AMP, IMPase, inositol monophosphatase, GSK3β, glycogen synthase kinase-3 β, I1R, imidazoline-1-receptor, SMERs, small molecule enhancers of rapamycin, SMIRs, small molecule inhibitors of rapamycin, Polyglutamine diseases, Autophagy, Neurodegeneration, Huntington's disease

## Abstract

In polyglutamine diseases, an abnormally elongated polyglutamine tract results in protein misfolding and accumulation of intracellular aggregates. The length of the polyglutamine expansion correlates with the tendency of the mutant protein to aggregate, as well as with neuronal toxicity and earlier disease onset. Although currently there is no effective cure to prevent or slow down the progression of these neurodegenerative disorders, increasing the clearance of mutant proteins has been proposed as a potential therapeutic approach. The ubiquitin–proteasome system and autophagy are the two main degradative pathways responsible for eliminating misfolded and unnecessary proteins in the cell. We will review some of the studies that have proposed autophagy as a strategy to reduce the accumulation of polyglutamine-expanded protein aggregates and protect against mutant protein neurotoxicity. We will also discuss some of the currently known mechanisms that induce autophagy, which may be beneficial for the treatment of these and other neurodegenerative disorders.

## Highlights

► The ubiquitin–proteasome system and autophagy are two main degradative pathways. ► Autophagy upregulation may protect against polyglutamine-expanded protein neurotoxicity. ► Autophagy compromise may occur in certain neurodegenerative diseases.

## 1 Introduction

Protein misfolding and subsequent aggregation are common features in many late-onset neurodegenerative disorders, such as Parkinson's disease, Alzheimer's disease and other tauopathies. The presence of these protein aggregates in brains of patients has been correlated with neuronal cell death and with earlier onset and increased symptom severity (Soto and Estrada, 2008). These disorders are commonly referred to as proteinopathies and include a group of conditions in which the aggregated proteins are encoded by genes containing trinucleotide repeat expansions. When this trinucleotide encodes the amino acid glutamine, it results in proteins with abnormally extended polyglutamine tracts and the disorders are hence termed poluglutamine disorders (Orr and Zoghbi, 2007). These expanded regions confer the protein the tendency to aggregate when the number of repeats exceeds a normal physiological number. Whether aggregated forms of these proteins and their intermediate forms represent toxic or protective species has been a matter of debate (Takahashi et al., 2010). However, the mutant proteins cause disease via a toxic gain-of-function mechanism, and it is generally accepted that degradation of polyglutamine-containing proteins would be a beneficial therapeutic approach for the treatment of these diseases. Two main degradative pathways are responsible for clearance of misfolded and unnecessary proteins in the cell: the ubiquitin–proteasome system (UPS) and autophagy (Rubinsztein, 2006). While oligomierised forms of proteins are inefficiently degraded by the proteasome, they can be targeted for degradation by autophagy, a lysosomal degradative pathway. In this review, we will focus on the role of autophagy in polyglutamine disorders, mainly Huntington's disease, the most prevalent of these conditions. We will review some of the increasing number of studies showing the potential benefit of upregulating autophagy for reducing the presence of these protein aggregates and therefore for the treatment of these and, other aggregate-prone protein disorders. We will also discuss different pharmacological approaches that, through autophagy stimulation, provide protection in polyglutamine neurodegenerative disorders.

## 2 Polyglutamine diseases

### 2.1 Genetics of CAG repeat disorders

Polyglutamine diseases consist of a group of ten autosomal dominant neurodegenerative disorders, which include Huntington's disease (HD), dentatorubral-pollidoluysian atrophy (DRPLA), spinal and bulbal muscular atrophy (SBMA), several types of spinocerebellar ataxias (SCAs), and the more recently proposed Huntington's disease-like 2 (HDL-2) (Orr and Zoghbi, 2007; Wilburn et al., 2011). Despite the large spectrum of neurological, psychiatric and motor symptoms present in these conditions, they all lead to chronic, slow progressive diseases affecting the central nervous system, for which no cure is available to date.

These disorders share a common genetic etiology, in which genes contain a repetitive DNA sequence consisting of the trinucleotide CAG, coding for the amino acid glutamine. This CAG rich region is unstable and tends to expand from one generation to the next (La Spada et al., 1994). As a consequence, the resulting protein contains an abnormal extension of polyglutamines that leads to individuals developing the disease when the repeats exceed a certain number. The threshold differs between diseases and is usually around 40 glutamines (Semaka et al., 2006; Langbehn et al., 2010). However, in the case of SCA6, an expansion between 18 and 33 glutamines in the CACNA1A gene, which encodes the alpha1A subunit of the P/Q-type voltage-gated calcium channel, is sufficient to cause the disease (Riess et al., 1997). Although in all known polyglutamine disorders there is a direct correlation between the severity of the disease and the polyglutamine length, the molecular and cellular mechanisms underlying the pathology are not fully understood (Orr, 2001). Moreover, the population of target neurons and the symptoms differ from one disease to the other, indicating that the nature of the affected protein as well as other genetic factors contribute to the progression of the disease and specificity (Gatchel and Zoghbi, 2005).

Proteins involved in CAG repeat disorders have crucial cellular activities, and are involved in different functions such as transcription, signalling or transport. And it is therefore possible that some aspects of the disease phenotype arise from a loss-of-function of the wild-type protein. However, mice heterozygous for Htt deletion do not mimic HD pathology, similar to the lack of evidence of ataxia or neurodegeneration in ataxin-1-null mice (Duyao et al., 1995; Zeitlin et al., 1995; Matilla et al., 1998). In contrast, experimental evidence suggests that that these diseases result mainly from a gain-of-function of the protein carrying a CAG expansion. Transgenic expression of the first exon or the full length Htt protein with an expanded polyglutamine produces pathological and phenotypic features of HD (Mangiarini et al., 1996; Hodgson et al., 1999). Moreover, a mouse model ectopically expressing a polyglutamine repeat presented a neurotoxic phenotype, characteristic of polyglutamine disorders, as well as the presence of intraneuronal protein aggregates (Ordway et al., 1997), suggesting that the polyglutamine repeat itself is sufficient to render neuronal cell death. A recent study has suggested that any contribution of a loss-of-function mechanism to HD may be minimal. Transcriptional regulation was compared between cells expressing a polyglutamine-expanded Htt and Huntingtin-null cells, and there was no overlap in the genes regulated in each condition, suggesting that a loss of the wild-type Htt does not contribute to the pathology of HD (Jacobsen et al., 2011).

### 2.2 Properties and toxicity of polyglutamine aggregates

Similar to other proteinopathies, the presence of intraneuronal protein aggregates is a common feature in the brains of patients suffering from CAG repeat disorders. Although the polyglutamine tract is common to all of these disorders and is responsible for the aggregation of the protein, the neuronal populations and brain areas targeted are protein-specific, suggesting an important contribution of the surrounding protein domains, posttranslational modifications, cleavage or cellular localization in their pathogenesis (Robertson and Bottomley, 2010). For instance, phosphorylation of threonine 3 in the first exon of mutant Htt protein increases its tendency to aggregate and cause pathology (Aiken et al., 2009). Aggregates, also termed inclusion bodies (IBs), can be observed intracellularly by light microscopy and are positive for components of the ubiquitin–proteasome system as well as chaperones. IBs present an amyloid structure similar to α-synuclein or Aβ peptide deposits, and this has been confirmed using anti-amyloid antibodies, thioflavin binding or Congo red birefringence. In the folding process between the soluble monomeric forms and the IBs, however, a variety of intermediate oligomeric forms have been described, including spherical or annular structures, amorphous forms, protofibrils and fibrils (reviewed in Hands and Wyttenbach, 2010).

The role of these protein aggregates in the disease is controversial. Whether aggregates or the intermediate forms represent toxic, protective or just incidental species is currently unclear – indeed, there may be multiple species mediating toxicity via distinct mechanisms. A protective role of protein inclusions, possibly by sequestering potentially more toxic intermediate oligomeric forms of the polyglutamine-containing protein, has been suggested in mouse models of HD (Arrasate et al., 2004), SCA1 (Watase et al., 2002) or SCA7 (Yoo et al., 2003). Along these lines, work from Takahashi et al. (2008) in neuronally differentiated cells showed that soluble oligomers of polyglutamines are more toxic than monomeric forms or the IBs. However, there is also evidence supporting a deleterious effect of protein inclusions. For instance, it has been shown that aggregate formation correlates with apoptotic cell death in cultured cells (Hackam et al., 1998; Lunkes and Mandel, 1998) and in a mouse model of HD (Yamamoto et al., 2000). In general, it is accepted that, although the nature of the toxic forms is not known, the propensity to aggregate is related to neuronal toxicity, which is supported by two facts: First, the length of the polyglutamine tract directly correlates with the tendency to aggregate, and second, the number of polyglutamines determines the severity of the disease and earlier age of onset (Perutz and Windle, 2001).

Consequently, decreasing the levels of expanded polyglutamine proteins has been pursued as a therapeutic strategy aimed to reduce the presence of toxic forms of aggregate-prone proteins. Strategies directed towards decreasing protein levels by affecting protein synthesis are being developed by targeting their expression using RNA interference. Since 2005, different HD mouse models, RNAi types and delivery methods have been tested with successful improvements in HD phenotypes and therefore this represents a promising human therapeutic strategy (Harper et al., 2005; Rodriguez-Lebron et al., 2005; Boudreau et al., 2009). However, several toxicity issues still need to be addressed related to the off-target effects of RNAi oligonucleotides and the effects of long-term treatments. Also, in the heterozygous state it needs to be considered what the negative implications of simultaneously silencing both mutant and wild-type genes are, as the consequences of this are not entirely understood (reviewed in Harper, 2009). An alternative strategy to avoid the accumulation of toxic proteins consists of accelerating their degradation, which, if specifically targeted to the mutant form of the protein, could represent a beneficial and safe therapeutic approach.

### 2.3 Mechanisms for clearance of polyglutamine proteins: the UPS and autophagy

In eukaryotic cells, two main pathways are necessary for degradation of misfolded proteins: the ubiquitin–proteasome system (UPS) and autophagy. In the former process, the 76 amino acid protein ubiquitin is conjugated to a lysine residue on the degradation-bound substrate via the concerted actions of three enzymes: an activating enzyme (E1), a conjugating enzyme (E2), and a ligase (E3). Through this pathway, soluble, short-lived proteins are targeted to the 26S proteasome, a multisubunit protease (Pickart, 2001).

Autophagy, on the other hand, does not necessarily involve selective cargo recognition but it degrades long-lived intracytoplasmic proteins and organelles by engulfment of portions of cytoplasm into a double-membrane vesicle called the autophagosome. The autophagosome then fuses with the lysosome and protein degradation occurs, as will be discussed in more detail in Section 3 (Ravikumar et al., 2010b).

Although Htt is ubiquitinated and targeted for degradation by the proteasome, Venkatraman et al. (2004) and Holmberg et al. (2004) showed that the polyglutamine tract cannot be efficiently cleaved by the catalytic subunit of the proteasome, thus releasing an isolated polyglutamine tract from the proteasome, which could constitute an even more aggregate-prone and toxic form.

The UPS is a degradative pathway that is constitutively active under normal conditions and thus it is difficult to find mechanisms for enhancing its activity. Recently, Lee et al. (2010a) described a non-catalytic approach to increase proteasome activity. They identified a small molecule that inhibited the deubiquitinating enzyme USP14, involved in ubiquitin chain trimming. Treatment with this compound enhanced degradation of proteins important in neurodegeneration such as tau, TDP43 or ataxin-3 (a polyglutamine expanded protein causing SCA3), and could provide novel strategies for increasing protein degradation by regulating protein degradation by the UPS. However, enhancing degradation by the proteasome may affect key short-lived proteins whose levels are tightly regulated, such as p53, and thus may have deleterious effects.

In the following sections, we will review the autophagic machinery and how upregulation of autophagy could be beneficial in HD and other polyglutamine diseases, as well as how autophagy homeostasis is compromised in these and other neurodegenerative diseases. We will finally comment on novel therapeutical strategies that have been proposed for these disorders based on an enhancement of autophagic activity.

## 3 Autophagy

### 3.1 Introduction

Macroautophagy is a bulk degradation process conserved from yeast to humans. Portions of cytoplasm are engulfed into a double-membraned vesicle, the autophagosome, which is degraded by subsequent fusion with lysosomes. While we will focus on macroautophagy, it is worth noting that other subtypes of autophagy exist. Chaperone-mediated autophagy degrades soluble cytoplasmic substrates containing a KEFRQ-like pentapeptide motif that is recognized by the chaperone heat shock cognate protein of 70 kDa (Hsc70). The substrate is targeted to the lysosomal membrane, where it interacts with lysosome-associated membrane protein type 2A (LAMP2A) and is unfolded before being translocated across the membrane for degradation within the lysosome (Kaushik et al., 2011). Microautophagy involves invaginations of the lysosomal membrane. A similar process, termed endosomal-microautophagy occurs in late endosomes and requires the action of endosomal sorting complex required for transport I (ESCRT-I), ESCRT-III and Hsc70 (Sahu et al., 2011).

**Fig. 1:**
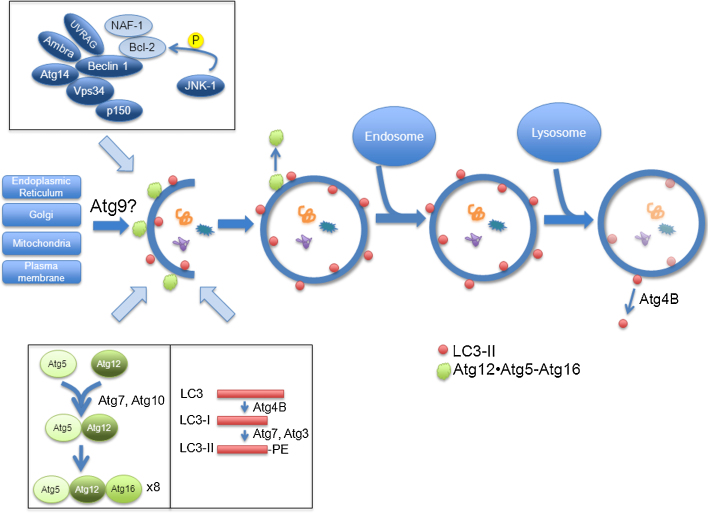
Autophagy machinery. Autophagy is a bulk degradation process in which portions of cytoplasm are engulfed by autophagosomes and degraded by fusion with lysosomes. Current evidence suggests that multiple compartments, including the endoplasmic reticulum, Golgi, mitochondria, and the plasma membrane may act as lipid donors to the growing phagophore, and Atg9 has been implicated in the membrane delivery. The Beclin 1 complex regulates the formation of autophagosomes, and includes autophagy-related proteins (shown in dark blue), and proteins that inhibit autophagy (shown in light blue). An important component of the complex is Vps34, a class III phosphatidylinositol 3-kinase responsible for the formation of phosphatidylinositol-3-phosphate, which is thought to recruit autophagy-specific proteins. The anti-apoptotic protein Bcl-2 inhibits autophagy, but is itself inhibited by phosphorylation mediated by JNK1 under starvation conditions. Two ubiquitin-like reactions contribute to the elongation of phagophores. In the first, Atg12 is conjugated to Atg5 via the concerted actions of Atg7 and Atg10 (E1-like and E2-like, respectively), and the resulting conjugate associates with Atg16L. This complex is found on the outer leaf of phagophores, and dissociates from completed autophagosomes. In the second ubiquitin-like reaction, LC3 is first trimmed by Atg4B to form LC3-I, and is subsequently conjugated to phosphatidylethanolamine by Atg7 and Atg3 to form LC3-II. LC3-II is found on the inner and outer membranes of phagophores and autophagosomes, and is recycled from the outer membrane of mature autolysosomes by Atg4B.

Macroautophagy (henceforth referred to as autophagy), on the other hand, begins with the formation of a phagophore or pre-autophagosomal structure, which elongates and fuses to form a double-membraned vesicle known as the autophagosome (Fig. 1). Autophagosomes can fuse with endosomes to form amphisomes (Gordon and Seglen, 1988), and eventually with lysosomes to form autolysosomes, where the contents are degraded by lysosomal hydrolases. This process is upregulated under starvation and stress conditions, where it functions to liberate nutrients. For example, immediately after birth, neonates face a period of starvation prior to receiving nutrients through milk. During this time, autophagy is significantly upregulated, and newborn mice deficient in autophagy fail to survive (Kuma et al., 2004).

Even in basal conditions, however, autophagy can work to clear misfolded proteins and damaged organelles. For instance, the autophagic degradation of mitochondria – termed mitophagy by Lemasters (2005) – protects against cell death by ridding the cell of damaged mitochondria and thereby preventing the production of excessive reactive oxygen species (ROS), the release of proapoptotic proteins such as cytochrome c, and subsequent activation of caspase 3 and 9 (Ravikumar et al., 2006; Rodriguez-Enriquez et al., 2006; Kim et al., 2007).

In addition to performing housekeeping functions, autophagy has several other functions, including the clearance of infectious agents, such as *Mycobacterium tuberculosis* (Gutierrez et al., 2004) and Group A *Streptococcus* (Nakagawa et al., 2004), aiding in antigen presentation via major histocompatibility complex class II (MHC II) (Lee et al., 2010b) and development (Cecconi and Levine, 2008).

### 3.2 Autophagy machinery

Studies in yeast have identified approximately 30 autophagy-related (ATG) genes required for autophagy, many of which have mammalian orthologues (Meijer et al., 2007; Xie and Klionsky, 2007). These genes are involved in various stages of the autophagy pathway: beginning with initiation of autophagosome formation, followed by the elongation of the membrane to a complete autophagosome, and ending with maturation – the fusion with lysosomes.

#### 3.2.1 Initiation

The formation of phagophores (the precursors of autophagosomes) begins at so-called phagophore assembly sites. The source of membrane for the nascent phagophore is still a topic of considerable debate. Some studies point to the endoplasmic reticulum (ER) as a contributor to the pre-autophagosomal structure: using various fluorescence imaging techniques, Axe et al. (2008) showed that autophagy-specific proteins accumulate at structures termed omegasomes that are associated with the ER, and eventually autophagosomes begin to appear. Later work using electron microscopy and 3D tomography supports this view (Hayashi-Nishino et al., 2009; Ylä-Anttila et al., 2009). Meanwhile, other groups suggest that the Golgi complex (Geng et al., 2010; van der Vaart et al., 2010) or mitochondria (Hailey et al., 2010) act as lipid donors. Recent work from our group has implicated the plasma membrane in contributing to the forming phagophore through clathrin-mediated endocytosis (Ravikumar et al., 2010a). It is quite possible that phagophores acquire membrane from different subcellular structures.

A critical protein regulating autophagosome formation is the class III phosphatidylinositol 3-kinase (PI3K) Vps34, the inhibition of which blocks autophagy (Blommaart et al., 1997) (Fig. 1). Vps34 is responsible for the formation of phosphatidylinositol-3-phosphate (PI-3-P). Regions in which PI-3-P is enriched may function to recruit autophagy-specific proteins and have been found to associate with forming phagophores (Axe et al., 2008). Vps34 is found in a complex with other autophagy-related proteins, including Beclin 1 (the mammalian orthologue of yeast Atg6), p150, Barkor/Atg14, UVRAG and Ambra 1 (Kihara et al., 2001; Itakura et al., 2008; Sun et al., 2008; Liang et al., 2008; Fimia et al., 2007). Under non-starvation conditions, the anti-apoptotic protein Bcl-2 binds to Beclin 1 and inhibits the formation of the Beclin 1/Vps34 complex, thereby inhibiting autophagy (Pattingre et al., 2005). Successful inhibition of Beclin 1 by Bcl-2 also requires nutrient-deprivation autophagy factor-1 (NAF-1), a small integral membrane protein in the ER (Chang et al., 2010). Upon starvation, however, c-Jun N-terminal protein kinase 1 (JNK1) phosphorylates Bcl-2 on multiple residues, causing it to dissociate from Beclin 1, and allowing for the activation of autophagy (Wei et al., 2008).

In another complex, the kinase ULK1 – which localizes to isolation membranes under starvation conditions – is also important for autophagosome biogenesis, as its depletion compromises autophagy (Chan et al., 2007). Its binding partners, FIP200 and Atg13, are responsible for the proper localization of ULK1 to isolation membranes, and furthermore stimulate its kinase activity (Ganley et al., 2009).

#### 3.2.2 Elongation

While the membrane source for the growing phagophore remains unclear, Atg9 – the only transmembrane Atg protein – has been observed to cycle between the trans-Golgi network (TGN) and endosomes (Young et al., 2006). Upon starvation, Atg9 redistributes from the Golgi near the nucleus to the periphery, where it colocalizes with LC3 and Atg16L, leading to speculation that Atg9 might function to deliver membrane during phagophore formation expansion. This process requires ULK1, PI3K activity, and the membrane curvature-driving protein Bif-1 (Takahashi et al., 2011; Young et al., 2006).

The elongation of phagophores requires two ubiquitin-like reactions (Fig. 1). In the first reaction, the ubiquitin-like molecule Atg12 is conjugated to Atg5 via an isopeptide bond through the actions of Atg7 (E1-like enzyme) and Atg10 (E2-like enzyme) (Mizushima et al., 1998, 2002; Tanida et al., 2001). This conjugate associates with Atg16L and oligomerizes to form a ∼800 kDa complex (Mizushima et al., 1999, 2003). The interaction of Atg5 and Atg16L is required to target the complex to autophagosome precursors, and the presence of all three proteins is required for the elongation of the isolation membrane. The Atg12–Atg5·Atg16L complex localizes to the outer membrane of elongating phagophores, but dissociates from complete autophagosomes (Mizushima et al., 2003).

In the other ubiquitin-like reaction, microtubule-associated protein 1 light chain 3 (MAP1-LC3, or simply LC3), the mammalian orthologue of Atg8, is conjugated to phosphatidylethanolamine (PE). First, the C-terminus of pro-LC3 is cleaved by Atg4B to expose a conserved glycine residue and thus form LC3-I, a cytosolic form of the protein (Hemelaar et al., 2003). Next, Atg7 acts as an activating enzyme to form an intermediate with LC3-I (Tanida et al., 2001), after which LC3 is transferred to the active-site cysteine of the E2-like enzyme Atg3 and is then conjugated to PE to form membrane-bound LC3-II (Tanida et al., 2002). LC3-II associates specifically with autophagosome membranes and remains bound even after fusion with lysosomes (Kabeya et al., 2000). In yeast, as well as in mammals, LC3 has been found to promote membrane tethering and fusion, suggesting that it enables the growth and expansion of the forming phagophore (Nakatogawa et al., 2007; Weidberg et al., 2011). Furthermore, LC3-II levels correlate with the number of autophagosomes present in the cell, making it the basis for many assays used in autophagy research (Kabeya et al., 2000; Klionsky et al., 2008; Rubinsztein et al., 2009). Although LC3-II is found on both sides of the autophagosome membrane, the molecules on the outer face are eventually delipidated by Atg4B and recycled (Tanida et al., 2004).

There is significant cross-talk between the two ubiquitin-like conjugation systems. In addition to the fact that the Atg16L complex brings LC3 to the site of lipidation and acts as an E3 for LC3-II conjugation (Fujita et al., 2008), Atg3 also facilitates the formation of the Atg12–Atg5 conjugate (Tanida et al., 2002). Meanwhile, Atg10, the E2-like enzyme in Atg12–Atg5 conjugation, also facilitates the conversion of LC3 to the lipidated form, although LC3 is not a substrate of Atg10 (Nemoto et al., 2003).

#### 3.2.3 Maturation

In the final steps of the autophagic pathway, autophagosomes may fuse with endosomes, forming amphisomes (Gordon and Seglen, 1988; Berg et al., 1998), and ultimately with lysosomes, forming autolysosomes. To achieve this fusion, autophagosomes move along microtubules towards lysosomes clustered at the center of the cell using the dynein–dynactin complex (Ravikumar et al., 2005; Jahreiss et al., 2008; Kimura et al., 2008). In fact, our group has found that the position of lysosomes themselves changes according to fluctuations in intracellular pH, driven by nutrient signalling. Starvation leads to increased intracellular pH, which enhances lysosomal clustering at the perinuclear area, putting them in the path of incoming autophagosomes and thereby facilitating autophagosome–lysosome fusion (Korolchuk et al., 2011). The machinery required for tethering and fusion includes soluble N-ethylmaleimide-sensitive factor attachment protein receptors (SNAREs) (Furuta et al., 2010). Finally, in order for the autophagosome cargo to be degraded, lysosomal function is also essential. The macrolide antibiotic bafilomycin A1 inhibits the lysosomal proton pump and thus prevents acidification of lysosomes. According to electron micrograph data by Yamamoto et al. (1998), which was later confirmed with fluorescent microscopy (Jahreiss et al., 2008), this also blocks the fusion of autophagosomes and lysosomes. In contrast, depletion of the syntaxin-5 SNARE complex impairs the anterograde trafficking of lysosomal proteases such as cathepsins, and while it does not affect fusion of autophagosomes with lysosomes, it does block their degradation (Renna et al., 2011).

### 3.3 Signalling pathways regulating autophagy

**Fig. 2:**
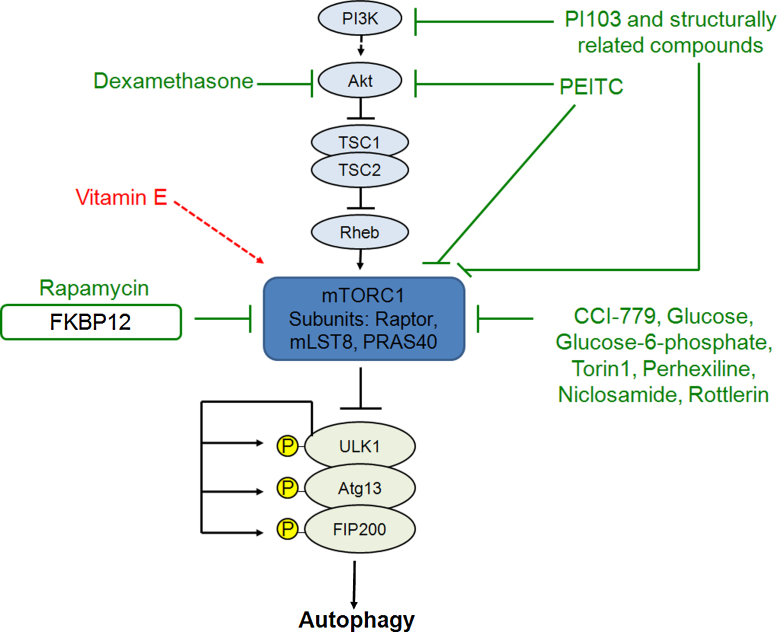
Inducing autophagy by inhibiting the mTOR pathway. mTOR is a downstream effector of the class I phosphoinositol 3-kinase (PI3K) pathway. The PI3K pathway regulates AKT phosphorylation which, in turn, inhibits the tuberous sclerosis complex (TSC)1/2, which activates the small GTPase Rheb, resulting in mTORC1 activation. Rapamycin interacts with FKBP12 which binds to and inhibits mTORC1. Inhibition of mTORC1 by rapamycin results in dephosphorylation-dependent activation of ULK1 and subsequent ULK1-mediated phosphorylation of Atg13, FIP200 and ULK1 itself, inducing autophagosome synthesis. The rapamycin analogue CCI-779, glucose, glucose-6-phosphate, Torin1, perhexiline, niclosamide and rottlerin also inhibit mTORC1 activity, either directly or indirectly. Dexamethasone induces autophagy via Akt inhibition. PI103 and structurally related compounds induce autophagy by inhibiting both PI3K and mTOR. Phenethyl isothiocyanate (PEITC) induces autophagy partially by suppressing the phosphorylation of Akt and mTOR. The vitamin E antioxidant activates the mTOR pathway and inhibits autophagy.

Several signalling pathways tightly control autophagy. The most well-known pathway involves the mammalian target of rapamycin (mTOR) complex (Fig. 2), which inhibits autophagy, but mTOR-independent pathways have also been recently characterised.

mTOR, isolated by Sabers et al. (1995), is a large serine/threonine protein kinase that exists in two complexes that comprise the mTOR pathway. Its canonical role is the regulation of cell growth and proliferation in response to nutrient conditions, but mTOR also regulates a variety of other cellular pathways, including autophagy, metabolism, translation, and ribosome biogenesis (Sarbassov et al., 2005; Loewith et al., 2002). The mTOR pathway involves two protein complexes, both of which contain mTOR and GβL (G protein β-subunit-like protein, or mLST8), but differ in their other subunits (Sarbassov et al., 2005). The rapamycin-sensitive complex, mTORC1, includes the protein raptor and is thought to respond to nutrient conditions and regulate autophagy (Sarbassov et al., 2005; Kim et al., 2002). Nutrient deprivation and rapamycin inhibit mTORC1 activity (Kim et al., 2002). The rapamycin-insensitive complex, mTORC2, contains rictor (also known as mAVO3), and has been shown to signal to the actin cytoskeleton, regulating actin polymerization and cell spreading (Jacinto et al., 2004).

A wide assortment of upstream signals affects the mTOR pathway, including glucose, amino acids and growth factors (Sarbassov et al., 2005) (Fig. 2). Many of these, however, converge on the tuberous sclerosis genes TSC1 and TSC2, which produce the proteins hamartin and tuberin, respectively, and act as regulators of mTORC1 (Tee et al., 2002). They have an inhibitory effect on mTORC1 via the small GTPase Rheb, for which TSC2 acts as a GTPase activating protein (Zhang et al., 2003). Under nutrient-rich conditions, for example, insulin binds to cell-surface receptors, activating the class I PI3K pathway, which catalyzes the conversion of phosphatidylinositol-4,5-bisphosphate (PIP_2_) to phosphatidylinositol-3,4,5-trisphosphate (PIP_3_) (Petiot et al., 2000). PIP_3_ then recruits proteins containing pleckstrin homology domains, including Akt and PDK1, the latter of which contributes to phosphorylating Akt (Lawlor and Alessi, 2001). Akt, in turn, inhibits the TSC1/TSC2 complex, allowing the activation of mTORC1 (Sarbassov et al., 2005). In contrast, when the nutrient supply is low, the AMP/ATP ratio increases, and AMP-activated kinase (AMPK) becomes active, phosphorylating and thereby enhancing the function of TSC2 (Inoki et al., 2003). This consequently leads to the inhibition of mTORC1 and the induction of autophagy.

In recent years, several groups have bridged the gap between mTOR and autophagy regulation by finding that the ULK1–Atg13–FIP200 complex is a downstream target of mTOR (Hosokawa et al., 2009). Under nutrient-rich conditions, mTORC1 associates with the complex and phosphorylates ULK1 and Atg13 (Hosokawa et al., 2009). The ULK1–Atg13–FIP200 complex is essential for autophagosome formation (Chan et al., 2007, 2009), and thus this inhibitory phosphorylation suppresses autophagy (Hosokawa et al., 2009). Recently, an mTOR-independent pathway for activation of ULK1 has been reported. Direct phosphorylation of ULK1 by AMPK in response to nutrient depletion leads to ULK1 activation and autophagy induction (Kim et al., 2011; Egan et al., 2011).

In addition to the canonical mTOR pathway, autophagy can be regulated via mTOR-independent mechanisms as well. For example, our laboratory has elucidated a cyclical pathway involving inositol and myoinositol-1,4,5-triphosphate (IP_3_), both of which inhibit autophagy (Sarkar et al., 2005). Intracellular cAMP acts (by means of Epac, Rap2B, and subsequently PLC-ɛ) to increase IP_3_ production (Williams et al., 2008). IP_3_, in turn, binds to receptors on the ER and leads to calcium release, which activates calpains that block autophagy (Williams et al., 2008). These pathways will be explained in greater detail in subsequent sections, as they were discovered and developed during the search for novel autophagy-modulating treatments.

## 4 Autophagy and neurodegeneration

Autophagy appears to be crucial to prevent neurodegeneration, even in the absence of disease-associated mutant proteins. Two independent studies using knockout mice for Atg5 or Atg7, found that the impaired autophagy function led to the accumulation of ubiquitin-positive inclusions and to the development of characteristic neurodegeneration phenotypes in these mice (Hara et al., 2006; Komatsu et al., 2006). In the presence of toxic proteins, autophagy upregulation has also been shown to be beneficial. In Alzheimer's disease, the Aβ peptide and the amyloid precursor protein (APP)-derived fragment (APP-CTF) are cleared upon autophagy induction (Tian et al., 2011).

Old or damaged mitochondria are less efficient in producing ATP and release greater amounts of reactive oxygen species (ROS), the main source of oxidative stress in the cells, and one of the hallmarks of neurodegeneration. Decreased autophagic degradation of mitochondria could therefore also constitute a key element in neurodegenerative diseases. In this context, two proteins related to autosomal recessive parkinsonism, PINK1 and Parkin, are important in mitochondrial homeostasis and have been recently implicated in the selective clearance of mitochondria by autophagy (Narendra et al., 2008, 2010). Thus, in Parkinson's disease, alterations in PINK1 or Parkin may contribute to the accumulation of dysfunctional mitochondria.

## 5 Autophagy implications in polyglutamine disorders

### 5.1 Polyglutamine-expanded proteins are substrates for autophagy

Autophagy was known since the early 1960s as a self-digestion process necessary for the bulk degradation of cytoplasmic content in lysosomes. It was not until 40 years later that autophagy was implicated in the degradation of aggregate-prone proteins and neurodegeneration. Some initial observations, such as the association of Htt protein with vacuoles presenting autophagosomal and autolysosomal features in a polyglutamine length-dependent manner, suggested a possible link between autophagy and neurodegenerative diseases (Kegel et al., 2000).

Later on, in 2002, we proposed that upregulation of autophagy could constitute a mechanism to prevent accumulation of aggregate-prone proteins (Ravikumar et al., 2002). We observed that chemical blockage of autophagy, by inhibition of autophagosome synthesis or disruption of autophagosome–lysosome fusion, increased the levels of mutant Htt and led to the accumulation of protein aggregates and to an exacerbation of the toxicity associated with these aggregates. This effect was not Htt-specific but it also reduced the number of aggregates of different aggregate-prone protein constructs consisting of purely expanded polyglutamines or polyalanines. Conversely, upregulating autophagy with the mTOR inhibitor rapamycin resulted in reduced aggregation and cytotoxicity (Ravikumar et al., 2002). Indeed, the benefit of rapamycin as a treatment of HD was confirmed *in vivo* in fly and mouse models, where administration of rapamycin or its analogues reduced the number of protein inclusions and improved motor and behavioural test performance in these HD models (Ravikumar et al., 2004). More recently, autophagy upregulation has been shown to be protective in zebrafish models of HD as well (Williams et al., 2008). The beneficial effects of rapamycin in these diseases are autophagy dependent, as it has no effects in proteionopathy fly models where there is reduced activity of autophagy genes (Berger et al., 2006; Pandey et al., 2007).

Genetic inhibition of autophagy mediated by silencing the expression of LC3 or Atg5 (Iwata et al., 2005) or Beclin 1 (Shibata et al., 2006) has supported the importance of autophagy in clearing polyglutamine-expanded proteins, including full-length mutant Huntingtin. Interestingly, chemical or genetic inhibition of autophagy has little or no contribution to the clearance of wild-type forms of Htt (Ravikumar et al., 2006). Moreover, an additional cytoprotective mechanism for rapamycin was proposed, as it protects cells and *Drosophila* against the toxicity of a range of pro-apoptotic insults. Although the mechanism remains unclear, a plausible explanation for the cytoprotective role of rapamycin involves mitochondrial clearance by autophagy causing a reduction in cytocrome c levels and caspase activation (Ravikumar et al., 2006).

Although initially studied in HD, the role of autophagy in clearing other cytoplamsic polyglutamine-expanded proteins is becoming more evident. For example, we have reported a beneficial effect of autophagy on clearance of ataxin-3, the protein responsible for SCA3, also known as Machado-Joseph disease, the most common type of SCA. Administration of a rapamycin analogue, CCI-779, to a SCA3 mouse model with an expanded ataxin-3 containing 70 glutamines (Bichelmeier et al., 2007), reduced soluble levels of expanded ataxin-3, decreased the number of aggregates in brains, and ameloriated motor dysfunction (Menzies et al., 2010).

In a recent study in a *Drosophila* model of DRPLA, where expression of a mutant form or atrophin-1 leads to neurodegeneration, a dramatic increase in the number of autophagosomal structures was observed (Nisoli et al., 2010). This suggested an alteration of autophagy regulation upon atrophin-1 expression, which was supported by an exacerbation of the atrophin-related neurotoxicity when autophagy was genetically impaired. However, when autophagy was upregulated by expression of a dominant-negative form of TOR or treatment with rapamycin, no rescue of the neurodegenerative phenotype in DRPLA flies was achieved. Further investigations of the autophagosome–lysosome structures found after expression of atrophin-1, showed an increase in the number of autophagic vesicles and autolysosomes. Although fusion between autophagosomes and lysosomes occurred normally, lysosomal degradation was impaired. This observation explains why induction of autophagy has no impact on aggregate clearance in DRPLA flies. Thus, in this specific polyglutamine expansion disorder, targeting the efficiency of the lysosomal degradation may be a more effective strategy for the treatment of DRPLA (Nisoli et al., 2010).

### 5.2 Molecular forms of polyglutamine-expanded proteins that are autophagy substrates

An important aspect in understanding the degradation of polyglutamine-expanded proteins by autophagy is to discern the molecular species that are targeted to autophagosomes for degradation. Whether autophagy reduces the accumulation of aggregates by engulfment of intermediate oligomeric forms or aggregated species, or whether aggregation is reduced by clearance of soluble proteins, thereby reducing the kinetics of the aggregation process, remains unknown. Although it has been reported that different autophagy proteins- LC3, Atg5, Atg12 or Atg16L- are recruited to cytoplasmic aggregates of polyglutamines and other aggregate-prone proteins (Iwata et al., 2005), it has not been observed that inclusions are directly engulfed by membranous vesicles. Furthermore, it should be noted that LC3 has been found to be recruited to polyglutamine aggregates in autophagy-deficient cells and, therefore, does not necessarily reflect the formation of autophagic structures (Kuma et al., 2007). Since autophagy substrates can be cleared in cell lines without inclusions visible by light microscopy, this suggests that autophagy can clear monomeric or oligomeric species (Webb et al., 2003) and perhaps the depletion of these leads to an indirect reduction of aggregate number.

Cellular distribution of protein aggregates is also a critical determinant when considering up regulation of autophagy as a therapeutic strategy for these conditions. The localization of these aggregates varies between different disorders. While in SCA1, SCA7, SCA17 and SBMA, aggregates accumulate in the nucleus, they are mainly cytoplasmic in SCA2 and SCA6, or are present in both locations in HD, SCA3, and DRPLA. This is important because cytoplasmic forms are degraded by autophagy, while autophagic clearance does not occur in the nucleus. Indeed, nuclear forms of expanded ataxin-1 are not degraded via autophagy, while a mutant form containing a defective nuclear localization signal was successfully cleared (Iwata et al., 2005). Similarly, in SBMA, the mutant androgen receptor binds to its ligand and is directed to the nucleus where it aggregates and induces toxicity within motor neurons. While autophagy can degrade cytoplasmic forms, it fails to clear the nuclear cytotoxic species (Montie et al., 2009). The fact that nuclear aggregates cannot be efficiently removed could explain the greater toxicity associated with intranuclear inclusions in diseases like HD where both cytoplasmic and nuclear aggregates are present (Yang et al., 2002).

Mutant Htt, as well as androgen receptor, ataxin-3, ataxin-7 or atrophin are targets for proteolytic cleavage by caspases, calpains, aspartic endopeptidases or, more recently identified metalloproteinases (Wellington et al., 1998, 2002; Miller et al., 2010), generating smaller N-terminal fragments containing the polyglutamine stretch. This proteolysis is associated with neurotoxicity and inhibition of caspases has been shown to be beneficial in different disease models of SCA3 (Berke et al., 2004), DRPLA (Ellerby et al., 1999), SCA7 (Garden et al., 2002) or HD (Graham et al., 2006). The presence of these truncated forms of the protein accelerates aggregation and influences its location. While longer forms of mutant Htt are localized mainly in cytoplasmic aggregates, small cleaved fragments have been shown to form both nuclear and perinuclear inclusions. Autophagy can degrade these N-terminal toxic forms as it has been reported for ataxin-7 (Mookerjee et al., 2009) or Htt (Ravikumar et al., 2002; Qin et al., 2003).

### 5.3 Selective degradation of protein aggregates by autophagy

Until recently, autophagy was considered a bulk protein degradation system that engulfed cytoplasmic content without apparent specificity. However, recent data indicates that some selectivity can enhance the degradation of damaged organelles, invading bacteria and certain misfolded proteins. For instance, mitochondrial degradation by autophagy in yeast has been recently shown to involve selective targeting by Atg32. Atg32 is a protein in the mitochondrial outer membrane that gathers other Atg proteins to damaged mitochondria in response to oxidative stress (Kanki et al., 2009; Okamoto et al., 2009). In higher eukaryotes, a similar cargo receptor function for mitophagy has been attributed to Nix/Bni3L (Schweers et al., 2007; Sandoval et al., 2008; Novak et al., 2010).

The ubiquitin-binding protein p62/SQSTM1 is recruited into ubiquitin-positive inclusions of tau and alpha-synuclein (Kuusisto et al., 2001), as well as into polyglutamine aggregates (Donaldson et al., 2003), and it has been suggested to function as a receptor for the selective autophagic degradation of ubiquitinated substrates. p62 contains an N-terminal PB1 domain for self-oligomerization and several domains for interacting with different proteins including ubiquitin, as well as LC3 (Komatsu et al., 2007; Ichimura et al., 2008). Inhibition of autophagy led to the accumulation of p62 inclusions, suggesting, together with the fact that p62 interacts with LC3, that p62 is an autophagic substrate. The self-oligomerization domain of p62 is required for its degradation as well as for the formation of protein inclusions. These data support a model in which p62 binds ubiquitinated proteins and through its self-oligomerization domain mediates their accumulation and aggregation in protein inclusions. p62 then binds and recruits LC3 to these aggregates which are degraded in an autophagy-dependent manner (Komatsu et al., 2007; Bjørkøy et al., 2005). While p62 depletion does not enhance mutant Htt accumulation (Korolchuk et al., 2009), it is possible there may be redundancy in this selective degradation system.

Alfy (autophagy-linked FYVE protein) is a PI-3-P binding protein that interacts with LC3 and is recruited to ubiquitin inclusions. Furthermore, it was proposed to target protein aggregates for degradation by autophagy (Simonsen et al., 2004). Later, it was shown that Alfy overexpression rescued polyglutamine toxicity in cells and *Drosophila* and that this effect is due to its function as an adaptor protein between p62 and autophagy effectors (Atg5, Atg12, Atg16L1 and LC3), facilitating autophagic degradation of p62-positive inclusions (Filimonenko et al., 2010).

The mechanisms described above require proteins to be polyubiquitinated prior to their degradation by autophagy. In contrast, a novel mode of selective degradation of mutant Htt involving previous posttranslational modifications by acetylation has recently been suggested. In this model, polyglutamine-expanded Htt is acetylated by CBP, which increases the rate of degradation of the protein. Although the exact mechanism remains unclear, the authors postulate that acetylation constitutes a signal to target mutant protein to autophagosomes for degradation (Jeong et al., 2009). Similarly, acetylation of lysine 257 in ataxin-7 by CBP and deacetylation by HDAC7 regulated the turnover of a cytotoxic fragment of ataxin-7 (Mookerjee et al., 2009). However, the consequences of this posttranslational modification were opposite to what was observed with mutant Htt. Ataxin-7 acetylation increased the stability of the protein and the polyglutamine length inversely correlated with this stabilization, providing a possible explanation for the higher toxicity of the polyglutamine-expanded protein. They suggested that autophagy is necessary for the clearance of the non-acetylated forms, this effect was disrupted in the presence of the autophagy inhibitor 3-MA but not by proteasome inhibition (Mookerjee et al., 2009). Further studies will be necessary to clarify the roles of different posttranslational modifications in the stability of these mutant proteins and their and degradation by autophagy in a selective manner.

### 5.4 Consequences of polyglutamine expansions on autophagic activity

As we have discussed, it has been extensively demonstrated that protein aggregation and cell toxicity can be slowed when autophagy is enhanced. However, whether accumulation of misfolded proteins is a consequence of autophagy dysregulation, or whether autophagy is upregulated in these disorders as a mechanism to counteract aggregate accumulation is not well understood. A connection between mutant Htt and autophagy alterations was described in a cellular model of HD, where expression of an expanded Htt in mouse striatal neurons was accompanied by an accumulation of membranes of the endosomal-lysosomal and autophagic system (Kegel et al., 2000). Also, wild-type Htt interacts with endosomal and autophagosomal membranes (Atwal et al., 2007) and with Rab5, which is involved in autophagosome formation (Ravikumar et al., 2008).

An upregulation of autophagy in the presence of polyglutamine aggregates was suggested by the finding that mTOR is sequestered into aggregates in cells expressing exogenous mutant Htt, HD mouse models and brains of HD patients, which results in the loss of its catalytic activity and should lead to enhanced autophagosome formation (Ravikumar et al., 2004). However, positive regulators of autophagy, such as Beclin 1, are also recruited into inclusions, with negative consequences for autophagy (Shibata et al., 2006). It is possible that autophagy levels change during the progression of the disease and during the different stages of the aggregation process. Recently, the generation of a knock-in mouse model with an early HD phenotype and early accumulation of inclusions generated by expressing a Htt protein with 200 glutamines presented an early activation of the autophagic response, suggesting that autophagy could be upregulated in the initial phases of HD (Heng et al., 2010).

A recent study has suggested a novel effect of mutant Htt in autophagy deregulation (Martinez-Vicente et al., 2010). While they did not observe impairment in autophagosome formation or in the levels of LC3-II in mouse embryonic fibroblast (MEFs) derived from an HD mouse model expressing the Htt protein with 111 polyglutamines, the rate of protein degradation in these cells was reduced. The authors found that autophagosomes from HD cells appeared relatively “empty” by electron microscopy and that the content of common autophagic cargo, such as mitochondria, polyubiquitinated proteins or lipid droplets, was reduced. Thus, the authors suggested that mutant Htt impairs cargo recognition by autophagosomes, which leads to a failure in protein degradation (Martinez-Vicente et al., 2010). How the polyglutamine expansion affects cargo recognition and whether this effect is Htt-specific or could be extended to other polyglutamine diseases and other proteinopathies requires further studies.

In an attempt to investigate the contribution of the polyglutamine expansion in disease, mice were generated where the normal 7 glutamine repeat stretch in murine htt was replaced with a mouse allele where the normal polyglutamine tract was eliminated (ΔQ-Htt) (Zheng et al., 2010). Deletion of the polyglutamine stretch rescued the HD phenotype caused by an expanded allele in trans, shown by a reduction in the number of mutant Htt aggregates, improved performance in motor and behavioural tests, and extended the life span. This longevity effect was observed not only when expressed in an HD model, but also in wild-type mice, expressing ΔQ-Htt. We also found that these mice had an increase in autophagy and that expression of ΔQ-Htt, but not the wild-type Htt, enhanced the clearance of aggregates when expressed in cultured cells. It seems, thus, that the polyglutamine stretch, even when expressed at its normal length, might have some negative effect, possibly by negatively regulating autophagy levels (Zheng et al., 2010).

## 6 Modulating autophagy to treat polyglutamine diseases

Given the protective effect that autophagy exerts in a variety of *in vitro* and *in vivo* models of polyglutamine diseases, the identification of strategies to induce autophagy might constitute a viable therapeutic approach to effectively treat these conditions. This can be achieved by enhancing autophagy via mTOR-dependent or mTOR-independent signalling, as has been shown with a range of autophagy modulators tested in various models of polyglutamine diseases (Table 1).

### 6.1 mTOR-dependent autophagy inducers

Until recently, the only drug known to upregulate autophagy was rapamycin, which induces autophagy by inhibiting mTOR. Therefore, rapamycin or its analogues were initially tested as an approach to enhance autophagic clearance of polyglutamine expanded proteins (Ravikumar et al., 2002, 2004). Other mTOR inhibitors have later been confirmed to induce autophagy (Fig. 2). Perhexiline, niclosamide, rottlerin and amiodarone were found in an automated cell-based assay screen of more than 3500 chemicals to induce autophagy via mTORC1 inhibition (Balgi et al., 2009). It should be noted that amiodarone was found in two independent screens of autophagy inducers. At lower concentrations, it induces autophagy in an mTOR-independent manner via Ca^2+^ channels, which will be reviewed below (Zhang et al., 2007; Williams et al., 2008). Also, torin1, a selective ATP-competitive small molecule has been reported to inhibit mTORC1 activity and subsequently increase autophagy to a much greater degree than rapamycin (Thoreen et al., 2009). As of yet, torin1 has not been tested in models of polyglutamine disease.

Increased intracellular glucose levels have been shown to enhance mutant Htt clearance and decrease mutant Htt aggregates. This effect is mediated by glucose 6-phosphate, which induces autophagy via mTOR inhibition (Ravikumar et al., 2003).

Autophagy induction via regulation of class I PI3K and Akt signalling has also been reported to be mediated by the glucocorticoid dexamethasone. In acute lymphoblastic leukaemia and multiple myeloma cells, dexamethasone induced autophagy through dephosphorylation and subsequent inactivation of Akt (Fig. 2) (Laane et al., 2009; Grandér et al., 2009). Phenethyl isothiocyanate (PEITC), an anti-cancer agent, has been suggested to induce Atg5-dependent autophagy. PEITC was found to increase autophagy partially due to its ability to suppress phosphorylation and activation of both Akt and mTOR (Bommareddy et al., 2009) (Fig. 2).

PI103 is a selective class I PI3K inhibitor that also inhibits mTOR in an ATP-competitive manner (Fig. 2) (Raynaud et al., 2007) and it has been shown to be a strong inducer of autophagy (Degtyarev et al., 2008). While PI103 itself cannot be used as an effective therapeutic approach due to its rapid *in vivo* metabolism and limited aqueous solubility (Degtyarev et al., 2008), it has been utilised to develop other dual PI3K and mTOR inhibitors (Liu et al., 2009a,b), which could have potential applications in treating some diseases.

It should be noted that antioxidants, such as vitamin E, have been considered as treatments for diseases such as HD to alleviate the oxidative stress that is commonly associated with the pathogenesis of neurodegeneration (Shoulson, 1998; Peyser et al., 1995; Kamat et al., 2008). Oxidative stress occurs when the production of ROS exceeds the capability of antioxidant mechanisms to effectively counterbalance ROS production. However, ROS are associated with the induction of autophagy and antioxidants can inhibit basal and induced levels of autophagy (Scherz-Shouval et al., 2007; Underwood et al., 2010). ROS scavengers block autophagy by increasing mTOR activity (Fig. 2), thus it is important to consider the effects that antioxidants may have in the induction of autophagy, specially because many HD patients take antioxidant active supplements.

As mTOR is a central regulator of many cellular processes (Sarbassov et al., 2005; Loewith et al., 2002) in addition to autophagy, mTOR-inhibition may have side-effects independent of autophagy that could limit its long-term compliance in diseases like HD. Rapamycin is an immunosuppressive agent and impaired wound healing and mouth ulceration are known side effects. Therefore, we and others have tried to identify mTOR-independent autophagy modulators.

### 6.2 mTOR-independent autophagy inducers

#### 6.2.1 Regulating the phosphoinositol pathway

**Fig. 3:**
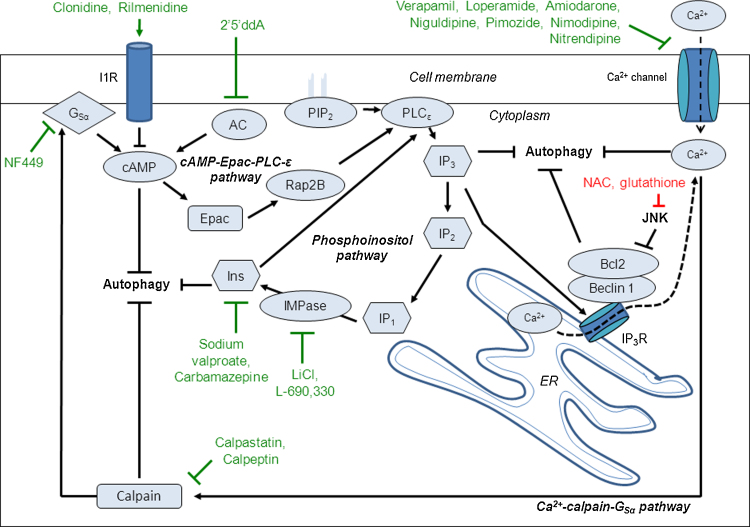
Inducing autophagy independent of the mTOR pathway. The cyclical mTOR-independent pathway consists of the cAMP-Epac-PLC-ɛ, phosphoinositol and Ca^2+^-calpain-G_Sα_ pathways and has multiple points where it can be modulated to induce autophagy in order to treat polyglutamine diseases. Intracellular cAMP levels are increased by adenylyl cyclase (AC), which activates Epac, which in turn activates the small G-protein Rap2B that activates phospholipase C (PLC)-ɛ. PLC-ɛ activation results in the production of IP_3_ from phosphatidylinositol 4,5-bisphosphate (PIP_2_) and IP_3_ binds to the endoplasmic reticulum (ER) IP_3_Rs releasing Ca^2+^ from ER Ca^2+^ stores. Intracytosolic Ca^2+^ levels are also increased by Ca^2+^ influx due to L-type Ca^2+^ channel agonist binding. Increase in intracytosolic Ca^2+^ activates the cysteine protease calpains which cleave and activate G_Sα_. G_Sα_ activation results in an increase in AC activity elevating cAMP levels, therefore as part of a loop. Activation of this loop pathway inhibits autophagy. Drugs targeting targets at different stages within the loop can induce autophagy and are protective in various polyglutamine disease models such as: imidazoline-1-receptor (I1R) agonists (clonidine and rilmenidine) and the AC inhibitor 2′,5′-dideoxyadenosine (*2*′*5*′*ddA*) that act to decrease cAMP levels; agents that reduce inositol and IP_3_ levels (lithium, L-690,330, sodium valproate and carbamazepine); Ca^2+^ channel blockers (verapamil, loperamide, amiodarone, nimodipine, nitrendipine, niguldipine and pimozide); calpain inhibitors (calpastatin and calpeptin) and the G_Sα_ inhibitor NF449. JNK phosphorylation of Bcl-2 results in the dissociation of Bcl-2 from Beclin 1 causing an induction of autophagy. The thiol antioxidants *N*-acetyl cysteine (NAC) and glutathione inhibit JNK activation and thus inhibit the phosphorylation of Bcl-2 resulting in the inhibition of autophagy as shown in cell, fly and zebrafish models of HD.

The first alternative mechanism that was characterised as increasing autophagy independently of mTOR was the reduction of intracellular levels of inositol or (IP_3_) (Sarkar et al., 2005). Mood-stabilizing drugs such as lithium, sodium valproate and carbamazepine, which reduce inositol levels (Williams et al., 2002), induced autophagic clearance of mutant Htt (Sarkar et al., 2005) (Fig. 3). Consequently, treatment with these drugs led to a reduction of mutant Htt aggregation in HD cell models, as well as alleviating the disease phenotype in fly models of HD (Sarkar et al., 2005, 2008; Williams et al., 2008).

Lithium inhibits several enzymes, including glycogen synthase kinase-3β (GSK3β) and inositol monophosphatase (IMPase) (Gould et al., 2002; Coyle and Duman, 2003). Lithium induces autophagy through the inhibition of IMPase, preventing inositol recycling downstream of IP_3,_ which was confirmed by using L-690,330, a specific IMPase inhibitor, that has a similar effect to lithium on the clearance of mutant proteins (Fig. 3).

Inositol-lowering drugs induce autophagy by reducing IP_3_ levels, since this effect is abolished by treatments that increase IP_3_ levels (Sarkar et al., 2005). IP_3_ can bind to IP_3_ receptors (IP_3_Rs) on the ER causing a release in Ca^2+^ from ER stores (Patterson et al., 2004) and elevated Ca^2+^ levels are known to inhibit autophagy (Gordon et al., 1993) (Fig. 3). As a consequence, autophagy can be induced through the pharmacological inhibition or genetic knockdown of IP_3_Rs (Fig. 3) (Criollo et al., 2007). Also, in the absence of IP_3_R, mitochondrial uptake of Ca^2+^ is reduced and leads to the activation of AMPK signalling and consequent induction of autophagy (Cárdenas et al., 2010). The activation of AMPK mediated by lower levels of Ca^2+^ is thus a very plausible mechanism accounting for the autophagy-inducing effects of agents reducing IP_3_ levels.

#### 6.2.2 Regulating the cAMP-Epac-PLC-ɛ pathway

In order to identify new mTOR-independent pathways to induce autophagy, we carried out a screen of 253 compounds comprising FDA-approved drugs and pharmacologically active compounds, analysing the effects of these drugs on the clearance of mutant Htt (Williams et al., 2008). Clonidine, an imidazoline-1 receptor (I1R) agonist, was identified in this screen as an mTOR-independent autophagy enhancer that increased clearance of mutant Htt. This drug, as well as rilmenidine (another clinically approved drug), enhances autophagy by lowering cAMP levels through its I1R agonist activity (Fig. 3) (Williams et al., 2008). Along the same lines, reducing cAMP levels by inhibiting adenylyl cyclase through 2′,5′-dideoxyadenosine also increased autophagy. cAMP regulates autophagy through Epac-PLCɛ signalling, which converges on the modulation of IP_3_ levels (Fig. 3).

Both clonidine and rilmenidine have been shown to induce autophagy and enhance the clearance of mutant Htt (Williams et al., 2008; Rose et al., 2010). Clonidine and 2′5′-dideoxyadenosine are protective in zebrafish models of HD and clonidine has also been shown to be protective in cells and flies expressing the mutant Htt protein (Williams et al., 2008). Recently, we reported the ability of rilmenidine to attenuate the disease phenotype in a mouse model of HD by reducing levels of mutant Htt fragments via the activation of autophagy (Rose et al., 2010). In safety trials, rilmenidine did not show an excess of adverse side effects when compared to placebo (Yu and Frishman, 1996). This indicates the possibility of using cAMP modulators to treat polyglutamine diseases, as many of them are already well-tolerated drugs used for the treatment of other conditions.

#### 6.2.3 Regulating the Ca^2+^-calpain-G_Sα_ pathway

In the same screen that identified clonidine as an autophagy enhancer, the L-type Ca^2+^ channel antagonists verapamil, loperamide, amiodarone, nimodipine and nitrendipine were identified to enhance autophagic clearance of mutant Htt proteins (Williams et al., 2008). When binding to L-type Ca^2+^ channels, these drugs prevent the influx of Ca^2+^ into the cell, and thus decrease the intracellular levels of Ca^2+^, resulting in increased autophagy, as previously reported (Fig. 3) (Gordon et al., 1993). The Ca^2+^ channel blockers niguldipine and pimozide (along with loperamide and amiodarone) were identified as autophagy enhancers in another screen analysing the effect of drug treatment on the number of GFP-LC3 vesicles in cells (taken to be a readout of autophagy) (Zhang et al., 2007). Rises in intracellular Ca^2+^ levels activate calpain activity, and calpain inhibition has also been shown in this screen to activate autophagy (Williams et al., 2008) (Fig. 3).

#### 6.2.4 Other mTOR-independent mechanisms

Trehalose, a dissacharide, is another mTOR-independent autophagy inducer (Table 1). It also acts as a chemical chaperone able to influence protein folding and aggregation through protein–trehalose interactions (Davies et al., 2006; Chen and Haddad, 2004; Sarkar et al., 2007a). Trehalose has been reported to reduce mutant Htt aggregation and toxicity in cell models of HD and attenuate disease pathology in a mouse model of HD via its chemical chaperone activity (Tanaka et al., 2004). Meanwhile, it can also enhance clearance of mutant aggregate-prone mutant Htt and protect against apoptotic insults in cells via its autophagy-inducing properties (Sarkar et al., 2007a). Thus, the additive effects of its autophagy-inducing and chemical chaperone activities, coupled with its lack of toxicity, suggest trehalose could be of potential benefit in the treatment of polyglutamine diseases.

A screen in yeast with 50,729 compounds was carried out to identify small molecule chemical modifiers of the cytostatic effects of rapamycin (Sarkar et al., 2007b). Small molecule enhancers of rapamycin (SMERs) and small molecule inhibitors of rapamycin (SMIRs) were tested in a secondary screen for their effects on autophagy that were independent of rapamycin. Three SMERs were identified as inducers of autophagy, which enhanced the clearance of mutant Htt fragments, reduced mutant Htt aggregation and were protective in cell and fly models of HD:SMER10, SMER18, and SMER28 (Table 1). The autophagy-inducing effects of these SMERs were indicated as being independent of mTOR, and 18 structural analogues were identified to also enhance the clearance of mutant Htt aggregate-prone proteins (Sarkar et al., 2007b).

Another screen identified fluspirilene and trifluoperazine (dopamine antagonists) and penitrem A (inhibitor of high conductance Ca^2+^-activated K^+^ channels) as autophagy enhancers (Zhang et al., 2007). They were found to reduce the number of expanded polyglutamine aggregates by increasing autophagy independently of mTOR (Table 1).

### 6.3 Combination treatment approaches that induce autophagy

Rapamycin or other mTOR inhibitors can be combined effectively with mTOR-independent inducers, such as trehalose, calpastatin and the SMERs, to enhance autophagic clearance of aggregate-prone proteins (Williams et al., 2008; Sarkar et al., 2007a,b). Combination treatment consisting of lithium or L-690,330 with rapamycin results in enhanced clearance of mutant Htt and enhanced protective effects in cell and fly models of HD, compared to treatment with either drug alone (Sarkar et al., 2005, 2008), These effects are due to the additive effects of mTOR inhibition (by rapamycin) and the mTOR-independent phosphoinositol pathway regulation (by lithium and L-690,330) in enhancing autophagy (Sarkar et al., 2005). Indeed, treatment of heterozygous TOR mutant flies (with impaired TOR activity) expressing mutant Htt with lithium shows higher neuroprotection when compared to non-treated heterozygous flies (Sarkar et al., 2008). In addition, lithium is also known to inhibit GSK-3β, which results in mTOR activation, which would inhibit autophagy (Inoki et al., 2006; Sarkar et al., 2008). Combination of lithium with rapamycin would help to counteract the undesired inhibition of autophagy resulting from GSK-3β activation (Sarkar et al., 2008). Lithium and rapamycin combination treatment of polyglutamine diseases is also attractive due to the additional protective effects that lithium GSK-3β inhibition has by exerting cytoprotective effects due to activation of the β-catenin/Tcf pathway (Carmichael et al., 2002; Berger et al., 2005; Sarkar et al., 2008).

These combination treatments are potentially desirable not only because of their additive effects in autophagic clearance of mutant proteins, but also due to the compensatory effect one drug may have for any unwanted side effects of the other drug that result from non-specific actions on alternative signalling processes. Therefore, one may be able to use lower doses of the respective drugs to achieve sufficient levels of autophagy induction, to achieve higher efficacy with minimal unwanted side effects. Further demonstration of combination strategies in animal polyglutamine disease models is required.

### 6.4 Future therapeutic prospects

Autophagy interacts with other cellular processes associated with neurodegeneration in polygutamine diseases and a greater understanding of this relationship is vital for determining the most effective treatment strategies. For example, the oxidative stress associated with HD can be targeted by antioxidant treatment in HD patients but, as we have shown, antioxidants can inhibit the basal and induced levels of autophagy (Underwood et al., 2010). Therefore, it is important to further test combinations of autophagy-inducing modulators with other polyglutamine disease treatments targeting different cellular processes associated with disease pathology.

In addition to this, it is obvious that early drug administration is key to effective treatment. Indeed, with monogenic diseases like polyglutamine diseases, most cases will have a family history and thus it is possible to treat patients at risk with pre-symptomatic treatment. Thus, better understanding of how diseases affect autophagy and how autophagy modulators may benefit disease may have clinical impact.

## Figures and Tables

**Table 1:**
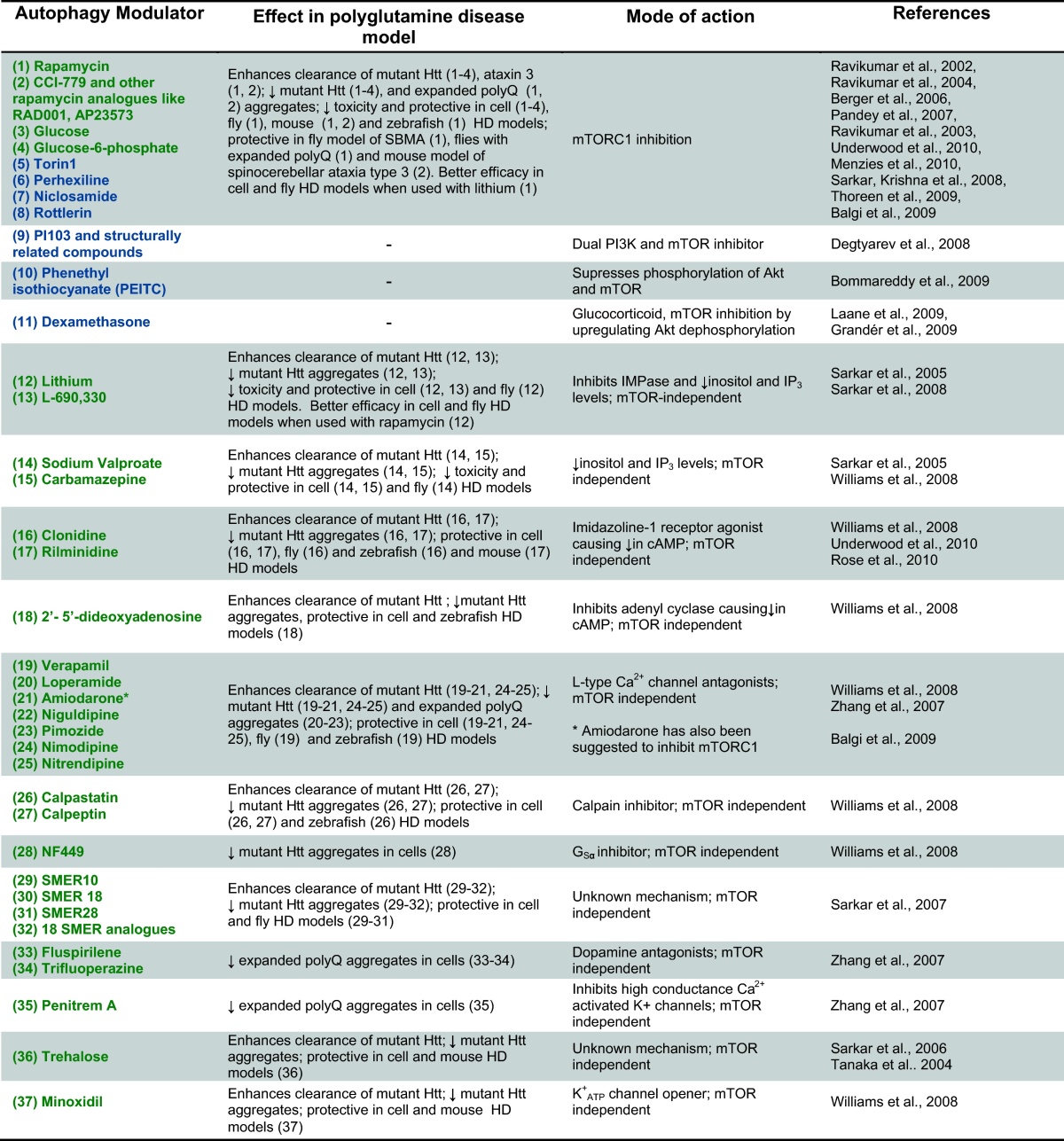
Autophagy modulators shown to be protective in polyglutamine disease models and their mode of action in modulating autophagy.

In green, autophagy inducers; in blue, autophagy modulators to be investigated further in polyglutamine disease models; ↑, increase; ↓, decrease.
